# Posttranslational Modifications Pattern in Clear Cell Renal Cell Carcinoma

**DOI:** 10.3390/metabo11010010

**Published:** 2020-12-27

**Authors:** Corina Daniela Ene, Mircea Nicolae Penescu, Simona Roxana Georgescu, Mircea Tampa, Ilinca Nicolae

**Affiliations:** 1 Faculty of General Medicine, Carol Davila University of Medicine and Pharmacy, 020021 Bucharest, Romania; mirceapenescu4@gmail.com (M.N.P.); simonaroxanageorgescu@yahoo.com (S.R.G.); tampa_mircea@yahoo.com (M.T.); 2 Carol Davila Clinical Hospital of Nephrology, 010731 Bucharest, Romania; 3 Victor Babes Clinical Hospital of Tropical and Infectious Diseases, 030303 Bucharest, Romania; drnicolaei@yahoo.ro

**Keywords:** posttranslational modification, renal cell carcinoma, cytokines, glycosylation, carbonylation, nitrosative stress, thiol/disulfide equilibrium, methylation, phosphorylation, protein cleavage

## Abstract

Posttranslational modifications are dynamic enzymatic-mediated processes, regulated in time and space, associated with cancer development. We aimed to evaluate the significance of posttranslational modifications in the pathogenesis of clear cell renal cell carcinoma. The authors developed a prospective, observational study during a period of three years and included 55 patients with localized renal cell carcinoma and 30 heathy subjects. Glycosylation, nitration and carbonylation, thiol-disulfide homeostasis, methylation, phosphorylation and proteolytic cleavage were evaluated in the serum of the evaluated subjects in the present study. Our results showed some characteristics for early ccRCC: high production of cytokines, substrate hypersialylation, induced nitrosative and carbonylic stress, arginine hypermethylation, thiol/disulfide homeostasis (TDH) alteration, the regulatory role of soluble receptors (sRAGE—soluble receptor for advanced glycation end products, sIL-6R—soluble receptor for Interleukin 6) in RAGE and IL-6 signaling, the modulatory effect of TK1—thymidine kinase 1 and TuM2-PK—tumoral pyruvate-kinase 2 in controlling the level of phosphometabolites in neoplastic cells. These data could be the initial point for development of a panel of biomarkers such as total sialic acid, orosomucoids, nitrotyrosine, carbonylic metabolites, Asymmetric Dimethylarginines (ADMA), Symmetric Dimethylarginines (SDMA), and thiol-disulfide equilibrium for early diagnosis of ccRCC. Moreover, they could be considered a specific disease posttranslational modification signature which underlines the transition from early to advanced stages in this neoplasia, and of a therapeutic target in kidney oncogenesis.

## 1. Introduction

Genetic and epigenetic alterations are considered primary steps in cancer development, while posttranslational modifications (PTMs) play an important role in cancer progression and dissemination. More precisely, posttranslational changes are dynamic enzymatic-mediated processes, regulated in time and space. From all these, glycosylation, carbonylation, thiol-disulfides status, glutathionylation, methylation, carbonylation, phosphorylation, and proteolytic cleavage have a major impact in tumorigenesis [1,2].

Glycosylation is a complex process, mediated by glycosyltransferases, which consists of covalent binding of hydrocarbonate to lipids or proteins. Glycobiology showed congenital alterations of glycosylation in autoimmune diseases, in diabetes, and in glomerulopathies [3].

In oncogenesis, glycosylation mediates cells adhesion, cells mobility, molecular traffic, cellular signaling; the ability of cancer cells to bypass cellular division checkpoints, to evade death signals and immune surveillance, to migrate to metastatic sites; to mediate immunity and clearance recognition [2,3,4,5]. Some studies showed aberrant glycosylation in patients with renal cell carcinoma (RCC) and alterations of N-glycosylation according to the stage of the disease [5]. Other studies analyzed the glycosylation of proteins involved in lipids transport and different metabolic processes (folate receptor 1—FOLR1; low density lipoprotein-related protein 2—LRP2; phospholipid phosphatase—PLPP; cathepsin D—CATD), in immune processes (CD97, CD63, CD276, anti-helicobacter pylori, alpha-1-antitripsine phenotype, prostaglandin D2 synthetase) [5,6,7,8,9,10,11].

Another characteristic of cancer is the imbalance between the increased level of oxidative stress and a high level of antioxidants, with the generation of increased reactive oxygen species [12]. Oxidative cleavage of proteins by either the α-amidation pathway or by the oxidation of glutamyl side chains can lead to the formation of oxidized amino acids and altered amino acid side chains containing reactive carbonyls [13]. Protein carbonylation is an irreversible posttranslational modification, responsible for the loss of protein function, as well as a trigger of specific signaling pathways involved in redox homeostasis [14,15]. Protein carbonylation was intensively studied in kidney disorders during recent years and their presence was associated with chronic kidney diseases [16,17], diabetic nephropathy [18], and atherosclerosis-mediated diseases [19,20], but no studies could be found in the literature about their implication in ccRCC. A marker of nitrosative stress by reactive nitrogen species production is nitrotyrosine (3-NT). It is a major product of peroxynitrite attack on proteins with an addition of a nitro group to the orthoposition of tyrosine. 3-NT has been used as a biomarker of nitrative damage in vivo [21]. The presence of 3-NT was previously reported in some carcinomas [22], in ccRCC through immunohistochemically staining, in contrast nephropathy [23], as a biomarker for vascular involvement in Fabry disease [24], and in preeclampsia [25].

Recent studies have demonstrated that functional thiol groups play a major antioxidant role that protects cells and tissue from oxidative damage. A dynamic thiol/disulfide equilibrium is pivotal in organizing antioxidant protection, xenobiotics detoxification, apoptosis, enzymatic regulation, transcription, cell division, cell growth, immune response, signal transduction, and cellular signal-transfer mechanisms [26,27,28]. The balance between the serum levels of thiol-proteins (albumin, cysteine, cysteinyl-glycine, glutathione, homocysteine, γ-glutamyl-cysteine-albumin) and the disulfides plays a protective role in cellular redox homeostasis [26,29]. In the literature, abnormal thiol/disulfide homeostasis was not evaluated in cancer patients, but it was associated with urolithiasis, hemodialysis, peritoneal dialysis, renal transplantation patients, acute renal failure, chronic kidney disease, obstructive uropathy, autosomal dominant polycystic kidney disease, urinary tract infections, and malignancy [28,29,30,31,32,33,34,35].

Protein methylation is a common posttranslational modification involved in many cellular processes and pathways, like cell transduction, signaling pathways, transcription, DNA repair, mRNA splicing, and tumorigenesis [36]. Arginine methylation as a PTM was intensively studied during the last few years due to recent discoveries about its involvement in cancer progression [37]. Asymmetric dimethylarginine (ADMA) and symmetric dimethylarginine (SDMA) amino acids are formed by protein arginine N-methyltransferases from methylated proteins on arginine residues and they are considered inhibitors of nitric oxide [38]. ADMA was first studied, while SDMA was considered an inert molecule, being first isolated from human urine [39]. These methylarginines are metabolized by two enzymes—dimethylarginine dimethylaminohydrolase-1 (DDAH-1) and -2 (DDAH-2)—as well as alanine-glyoxylate aminotransferase 2 (AGXT2). Some studies showed that both ADMA and SDMA have pro-inflammatory effects, and their levels are associated with cardiovascular risk and with chronic kidney disease progression [40,41].

Advanced glycation end products (AGE) are proteins or lipids that become glycated as a result of exposure to sugars. Kidney AGE formation is stimulated by oxidative stress, uremia, hyperglycemia, and the renin–angiotensin system. The receptor for advanced glycation end products (RAGE) is a multi-ligand cell surface protein [42,43]. AGE binding to RAGE-specific receptors, expressed on the renal, endothelial, immune, and inflammatory cells membrane, releases profibrogenic and proinflammatory cytokines, generates intracellular hydrogen peroxide and oxygen, and inhibits nitric oxide production [44,45,46,47]. The physiopathological role of RAGE signaling was demonstrated in many inflammatory and malignant disorders. AGE–RAGE interaction promotes tumoral cells’ survival and proliferation by autophagy, apoptosis, and necrosis inhibition and NF-KB activation [45]. Low levels of serum sRAGE were associated with a bad outcome in different diseases, while elevated serum levels of sRAGE were detected in diabetes and end-stage renal disease [44].

The physiopathological significance of cytokine release was documented in malignant and inflammatory diseases [47,48,49,50,51,52]. IL-6 is a pleiotropic cytokine that interacts with its specific 80kDa receptor (IL-6R), a protein complex consisting of a ligand binding chain (CD126) and a signal transducer (gp130 or CD130). On target cells, IL-6 is associated with IL-6R (found on a limited number of cells) and gp130 (expressed on most human cells); the complex dimerizes and initiates the intracellular signaling. This process is called classic IL-6-mediated signaling, this pathway being involved in the protective, regenerative, and anti-inflammatory activity of this enzyme. In those cells that do not express IL-6R, IL-6 signalize by a protein aggregate, consisting of IL-6, a soluble component of the receptor (sIL-6R) and membrane gp130. This process is called trans-signaling and it is involved in the proinflammatory and prooxidant activities of cytokines. The soluble forms of IL-6R, namely sgp130 and sIL-6R released from the cell membrane by proteolytic cleavage, play a decisive role in regulating IL-6-mediated signaling. It is appreciated that sgp130 is the natural inhibitor of IL-6-mediated trans-signaling [53,54,55].

Phosphorylation–dephosphorylation represents reversible PTMs, important for many cellular processes in the kidney, such as the cell cycle, cellular increase, apoptosis, signaling, the biological activity of podocytes, glomerular filtration, cellular adhesion, cytoskeleton stability, integrity of the split diaphragm, actin polymerization, endocytosis, stability of stress fibers, and impaired cellular motility [56]. A disruption in the phosphorylation–dephosphorylation equilibrium of nephrin, podocin, CD2-associated protein, synaptopodin, and actin-4 was detected in many kidney diseases: chronic kidney disease, primary glomerulopathies, severe proteinuria, and glomerular basement membrane thickening [56,57,58,59,60].

Little data about the role of phosphorylation–dephosphorylation in ccRcc in the literature could be found. Kinase- and phosphotransferase-mediated phosphorylation was investigated in some studies by detecting thymidine kinase 1 (TK) and tumor N2 pyruvate kinase (TuM2-PK) activity [61,62,63]. TK was considered an enzyme involved in nucleic acids synthesis and an important proliferation marker in tumoral cells [64,65]. TK has two isoforms: *cytosolic TK-1* detected in the proliferation phase of the cellular cycle, its synthesis being associated with cellular division and restricted in the S phase of the cellular cycle; and *mitochondrial TK-2*, which is not correlated with the cellular cycle.

TK-1 was found mostly in serum and it was used in the detection of aggressive tumors and for monitoring the evolution and therapeutic response in patients with solid cancer. Its variation after repeated assessments was considered to show the remission or the relapse of the tumor [56,61,62,63]. Many tumoral cells are characterized by pyruvate-kinase overproduction [66,67,68,69,70]. Many isoforms of pyruvate-kinases were identified L-PK (liver, kidney), R-PK (erythrocytes), M1-PK (muscles, brain), and M2-PK (liver, tumoral cells). In normal cells, M2-PK is tetrameric and active, with high affinity for phosphoenolpyruvate. In tumoral cells, it is dimeric, inactive, with low affinity for substrate, induced by interaction with different oncoproteins. This isoenzyme catalysis irreversible phosphorylation between phosphoenolpyruvate and adenosine diphosphate, producing pyruvate and adenosine diphosphate. M2-PK acts as a protein kinase involved in gene transcription; it reprograms oxidative phosphorylation in aerobe glycolysis, promoting tumoral cells proliferation and migration [70]. TuM2-PK, a tumoral cytosolic protein, without organ specificity is released in the extracellular microenvironment during tumoral progression. TuM2-PK was determined in the blood of patients with renal cancer and other nephropathies (nephritis, diabetic nephropathy). The circulant level of this protein shows the activity and progression of the tumoral process and it is important in identifying metastatic disease, the tumoral relapse, and in monitoring therapeutic response [66,68,69,70].

Clear cell renal cell carcinoma (ccRCC) is the most frequent and studied kidney cancer. Some studies regarding PTMs in ccRCC were developed but only using immunohistochemical staining, and none in patients’ sera [5,6,61,66,67,68,69,70]. The aim of the present study was to determine the significance of posttranslational modifications in the pathogenesis of renal cell carcinoma. We hypothesized that protein glycosylation, nitration, carbonylation, alteration of thiol-disulfide homeostasis, methylation, proteolytic cleavage, and phosphorylation could be important physiopathological processes in the early renal cell carcinoma microenvironment.

## 2. Results

### 2.1. Protein Glycosylation in ccRCC

Protein glycosylation, evaluated by serum levels of sialic acid and orosomucoids, was statistically significantly higher in the renal cell carcinoma group compared with the control group (sialic acid: 114.50 ± 22.05 vs. 50.13 ± 3.66 mg/dL *p* < 0.001; orosomucoids: 5.72 ± 0.47 vs. 0.86 ± 0.14 g/L, *p* < 0.001) (Table 1). Sialic acid increased 2.28-fold and orosomucoids 6.65-fold compared to the controls.

### 2.2. Protein Oxidation in ccRCC

Posttranslational protein oxidation expressed by serum levels of 3-nitrothirosine and carbonylated proteins were significantly increased in the RCC groups compared to the control group: 3-nitrotyrosine by 2.96-fold (0.335 ± 0.072 vs. 0.135 ± 0.012 μmol/L, *p* < 0.001) and carbonylic groups by 1.98-fold (45.14 ± 7.80 vs. 22.70 ± 2.23 μmol/L, *p* < 0.001) (Table 2).

### 2.3. Thiol-Disulfide Homeostasis in ccRCC

Thiol-disulfide homeostasis, defined by PTM, native thiol (NT), total thiol (TT), disulfide (DS)/NT, DS/TT, and NT/TT, varied significantly between the two groups. Gamma-glutamyl transferase (GGT) had no significant variation between the two groups. NT, TT, DS and DS/NT, and DS/TT ratios were significantly higher, while NT/TT was significantly lower in the renal cell carcinoma group when compared with controls (Table 3).

### 2.4. Protein Methylation in ccRCC

Protein methylation evaluated by serum levels of SDMA and ADMA and the ADMA/SDMA ratio showed an increase of 2.72-fold in SDMA, 1.45 in ADMA, and a decrease of 1.73-fold in ADMA/SDMA ratio in the RCC group when compared with the control, with statistically significant variations (Table 4).

### 2.5. Proteolytic Cleavage in ccRCC

Protein cleavage expressed by serum levels of sIL6R and RAGE had no statistically significant variation between the two groups (sIL-6R 100.58 ± 10.21 vs. 102.47 ± 10.18 ng/mL, *p* > 0.05; sRAGE 1001.47 ± 145.27 vs. 1014.40 ± 89.59 pg/mL *p* > 0.05) (Table 5).

### 2.6. Phosphorylation in ccRCC

Phosphorylation, based on phosphate group addition, was investigated by assessment of the serum levels of TK-1 protein and TuM2-PK. The comparative analysis of TK-1 levels in the RCC and control groups showed no statistically significant differences (Table 6).

### 2.7. Interplay between Posttranslational Modifications and Inflammation in ccRCC

The analysis of posttranslational modifications variation in relation to inflammation markers (CRP, IL-6, and IL-8) was evaluated in the RCC group (Table 7). We found a moderate positive relation between protein glycosylation products and inflammatory markers: sialic acid and CRP (rho = 0.26, *p* = 0.02), IL6 (rho = 0.39, *p* = 0.01), and IL-8 (rho = 0.26, *p* = 0.02) also between orosomucoids and CRP (rho = 0.24, *p* = 0.04), IL6 (rho = 0.30, *p* = 0.01) and IL-8 (rho = 0.25, *p* = 0.04). The significant positive correlations between nitrotyrosine and IL-6 (rho = 0.24, *p* = 0.04) and carbonylic groups and IL6 (rho = 0.40, *p* = 0.03) show an important protein oxidation process in patients from the RCC group. The negative statistically significant relations between NT and CRP (rho = −0.84, *p* < 0.001), TT and CRP (rho = −0.76, *p* < 0.001), also IL-6 (rho = −0.18, *p* = 0.03), NT/TT and CRP (rho = −0.40, *p* = 0.02), and the positive statistically significant relations between DS/NT and CRP (rho = 0.40, *p* = 0.02) and DS/TT and CRP (rho = 0.40, *p* = 0.02) showed alteration of thiol-disulfide homeostasis in relation with inflammation in renal cancer. Concerning TK-1, the result of phosphorylation strongly correlated with CRP (rho = 0.38, *p* = 0.04) in the RCC group, and TuM2-PK moderated correlated with IL-8 (rho = 0.30, *p* = 0.06).

There were no significant correlations between protein methylation products (SDMA, ADMA), protein cleavage markers (sIL6R and sRAGE) and CRP, IL-6, and IL-8 in the RCC group. There were no significant correlations between the posttranslational modification protein markers and CRP, IL-6, and IL-8 in the control group. Graphical representations of the results can be found in Appendix A.

## 3. Discussion

Clear cell renal cell carcinoma (ccRCC) is the most common form of human kidney cancer. Histological and molecular analyses suggest that ccRCCs have significantly altered metabolism. No previous studies that focus on posttranslational modifications in the serum of patients with clear cell renal cell carcinoma are present in the literature. Limited data present the synthesis and expression of posttranslational modifications of proteins in the urine of patients with renal cell carcinoma. The present study showed that the serum protein PTM profile is representative for kidney oncogenesis.

Recent studies have documented aberrant glycosylation in driving cancer progression to different stages across a variety of cancer types [71]. Our study showed that glycosylation, quantified by important increases in the serum levels of sialic acid and orosomucoid, was clearly expressed in patients with early stages of RCC. Their moderate positive correlation with inflammation markers (CRP, IL-8, IL-6) sustains their role in cancer progression. Orosmucoid, a polymorph acute phase reactant with immunosuppressant properties and sialic acid, frequently used in lipid and protein glycosylation, could act as a protective factor for tumoral cells against immunological attack by modulating the inflammatory reaction and against angiogenic response [72,73,74,75,76]. Our results regarding change in protein glycosylation status in patients with early RCC could be considered a characteristic of early stages in kidney tumorigenesis, a glyco-signature of RCC.

One recent study [5] developed a proteomic research, investigating the urinary N-glycoproteome of clear cell renal cell carcinoma at different evolution stages and showed an up-expression of P13P1, CD97, COCH and a down-expression of haptoglobin, fibronectin, ceruloplasmin, apolipoprotein B, phospholipids transfer protein, H factor of complement according to the RCC stage. On the other hand, Sadim et al. showed an up-secretion of haptoglobin in another proteomic study in urine from patients with clear ccRCC [77]. These contradictory results could be explained by the diversity of the malign phenotype. Based on these data, we can discuss about an equilibrium between glycolyzed and un-glycolyzed forms of the same protein and between glycoforms inside the cells and those secreted by the cells, aspects that show a dynamic status in the early phase of renal tumorigenesis. The data presented before are documented by studies on cell lines: urinary tract-associated, embryonic renal cells (HEK 293), embryonic carcinoma (NTERA2), normal renal tube cells (RPTEC/TERT1), human carcinoma (cells 786-0), renal human cells (Cake-1), bladder cancer (RT4), prostate cancer (PC-3) [5].

There is growing evidence that ccRCC is a neoplasia with significant alteration of cellular redox homeostasis, with marked oxidative alteration of proteins [78,79]. In our study, we have evaluated protein carbonylation, the most popular biomarker for severe oxidative damage and nitrotyrosine, a marker of nitrosative stress. Our results showed a high oxidative process in patients with ccRCC compared with healthy subjects. The present study is the first one in the literature that evaluates protein carbonylation and 3-NT in serum patients with localized ccRCC. Some studies in the literature evaluated serum oxidative stress through different parameters such as total oxidant status, total antioxidant capacity, paraoxonase-1, aryl esterase, total thiol, catalase, myeloperoxidase, and ceruloplasmin in patients with different types of renal cell carcinoma (72.3% patients with ccRCC) in different evolution stages and none could find a marker to discriminate between patients with RCC and healthy people [80]. Other studies showed significantly increased reactive oxygen species and nitric oxide, and decreased glutathione activity in patients with renal cell carcinoma compared to normal subjects [81]. Nitrosative stress was also evaluated in ccRCC through immunohistochemical staining with an antibody to nitrotyrosine, and a higher concentration was found in high grade tumors [82]. Many studies in the literature present overexpressed nitrotyrosine activity, mostly detected by immunohistochemical analysis such as in esophageal squamous cell carcinoma [83], breast cancer [84], and colon cancer [85]. Identification of carbonylated proteins and nitrotyrosine and defining their roles in ccRCC may help the development of targeted therapeutic approaches toward mitigating oxidative damage of these proteins.

Our study showed the alteration of thiol/disulfide groups, with statistically significant lower levels of NT and TT cytokines in patients with pT1N0M0 ccRCC, and their association with high inflammation. The decreased levels of thiols could be explained by their transformation in disulfurs by oxidation. The level of serum disulfurs was significantly higher in patients with ccRCC versus the control group. The equilibrium of disulfides-thiols was deviated through disulfides production. These results suggest that the reduction in NT and TT could be linked with the loss of antioxidant capacity as a response to inflammatory stimuli in neoplasia. Though, TDH alteration was present in the early stages of renal carcinogenesis and it could play a role in ccRCC physiopathology as an oxidative stress marker. More studies are needed for evaluating its role in early or advanced ccRCC. TDH status was used to evaluate the severity of multiple renal and inflammatory diseases (urolithiasis, obstructive uropathy, urinary tract infections, autosomal dominant polycystic disease, acute renal failure, chronic kidney disease, hemodialysis, peritoneal dialysis, renal transplantation) [29,30,31,32,34,35,63,64,65].

In patients with advanced malignancies (advanced clear renal cell carcinoma, urinary bladder cancer, multiple myeloma), the studies showed reduced levels of serum NT and TT according to the loss of antioxidant capacity as a response to high oxidative stress, and the decrease in disulfurs as a result of high usage of sulfur atoms during tumoral multiplication [86]. Our study is the first in the literature that evaluated TDH status in patients with early stages of ccRCC. More studies are needed to clarify TDH modifications in different models of malignancies according to their progression and clinical complications.

Protein methylation was linked with carcinogenesis and metastasis in some studies [36]. In our study, we evaluated ADMA and SDMA as markers for protein methylation. ADMA and SDMA are listed as uremic toxins and have been increasingly recognized as putative toxic non-proteinogenic amino acids in a wide range of human diseases [87]. Little attention has been paid to the pathophysiological role of SDMA. Both ADMA and SDMA may induce reactive oxygen species and enhancement of NADPH-oxidase, being involved in the pathogenesis of CVD [88]. These molecules were investigated in chronic kidney disease (CKD) and end-stage renal disease (ESRD) [89]. Circulating ADMA levels are elevated in patients with CKD, before a reduction in glomerular filtration rate, while SDMA has been considered a marker of acute kidney injury [90,91]. Some mechanisms of inhibition of the expression and/or activity of DDAHs have been described like hyperglycemia, oxidative stress, and angiotensin II administration [37,92,93]. This is the first study that evaluated ADMA and SDMA in renal cancer. In our study, ADMA and SDMA were significantly increased in patients with localized ccRCC, while the ADMA/SDMA ratio was significantly lower compared with the control group, probably due to DDAHs inhibition by oxidative stress in the tumor microenvironment. ADMA could be involved in tumor growth and neoangiogenesis by regulating the concentration of nitric oxide and altering vascular endothelial cell growth factor production [94,95]. They induce CD11a, CD11b, and CD14 expression in monocytes and CD18 expression in granulocytes to enhance the differentiation and adhesion capacity of leukocytes to the endothelium [41]. A recent study showed that gastric cancer patients with high ADMA levels had poor prognosis and low survival rate [94]. Another study considered ADMA and SDMA as novel prognostic factors for all-cause mortality in patients with hematological malignancies [96]. More studies are needed for evaluating the role of ADMA and SDMA in ccRCC physiopathology.

In ccRCC, the pathophysiological significance of soluble forms of cell surface receptors has not been clarified. The soluble forms of RAGE (sRAGE) and IL-6R (sIL-6R and sgp 130), derived from cell surface membrane receptors, by proteolytic cleavage, mediated by MMP-9 and ADAM-10, can function as modulators of these ligands’ bioactivity. In this study, the determination of the circulating level of sRAGE in patients with ccRCC showed values similar to those obtained in the control. In addition, the serum levels of IL-6, IL-8, and CRP did not exert any influence on sRAGE production in patients with impaired renal function. These data show that sRAGE formation in the tumor or stromal microenvironment could be an intrinsic mechanism responsible for downstream inhibition of RAGE-mediated cellular signaling. The use of recombinant sRAGE to block RAGE activation has already been shown to inhibit tumor growth in various cell models [97]. An AGER1 membrane protein has recently been described that, in conjunction with sRAGE, attenuates the harmful effects of RAGE activation by modulating the absorption, degradation, and elimination of AGE and suppressing the inflammation and oxidative stress generated by RAGE [46]. sRAGE could interact with ligands, but AGE–RAGE activation is blocked [46,47].

The present study showed that serum levels of IL-6 and IL-8 determined in patients with early ccRCC were higher compared to the control. These results suggest the presence of an inflammatory process in the tumor microenvironment. The determination of sIL-6R did not show significant variations between the two groups. Inflammation detected in patients with ccRCC induces IL-6 synthesis, while serum levels of sIL-6R remain at a constant level sufficient to block trans-signaling. Data in the literature showed that systemic IL-6 interacts with the soluble components of the receptor and forms protein aggregates IL-6:sIL-6R and IL-6:sIL-6R:sgp130. Based on the results of our study, it can be considered that sIL-6R, in combination with sgp130, acts as a natural buffer that blocks the trans-signaling of IL-6 and maintains low levels of inflammation [53,55,98].

Limited data are presented in the literature about TK-1 use in patients with ccRCC. Our study is the first one in the literature that investigated the serum levels of TK-1 in patients with early stages of clear cell renal carcinoma. We observed no statistically significant variation of TK-1 serum levels between the ccRCC group and healthy subjects. Based on our results, the serum assessment of TK-1 in these patients had no relevance for early detection of this cancer [99]. Few studies evaluated the levels of TK and pyruvate-kinase before the surgical removal of the renal tumor, and showed statistically significant increased levels of these enzymes when compared with the control group, but not when compared with renal benign tumors. The variation of these markers was correlated with tumoral stage (T). The presence of tumoral necrosis was linked to high TuM2-PK and low TK-1. The determination of TuM2-PK and TK-1 before and after surgical removal of the tumor could indicate the recurrence of the neoplasia and could help to stratify patients in risk groups and establish an appropriate antitumoral treatment [99]. Lou et al. showed that serum level of TK-1 was not correlated with immunohistochemical expression of the enzyme in tumoral tissue [88]. The serum level of TK-1 varies according to tumoral stage and therapeutic response, with increase in remission and decrease in relapse [99,100]. It can be used for early detection of relapse, after surgical removal of the tumor. Corroborating our results and those in the literature, it seems that TK-1 has prognostic value, though it can be an independent predictor of relapse-free interval. [100]. The present study showed no significant increase in quantitative determination of TuM2-PK in patients with T1N0Mo ccRCC compared with healthy subjects. The plasmatic level of TuM2-PK in patients with ccRCC was correlated with IL-8 levels, data that suggest a possible interaction between these markers and tumoral neoangiogensis [101,102,103,104,105,106]. A recent report showed that the extracellular level of TuM2-PK in patients with localized renal cell carcinoma was 3.2-fold lower compared with patients with disseminated disease [107]. Some studies showed that TuM2-PK levels were significantly higher in renal cancer patients compared with healthy subjects, revealing a strong relation between this marker and tumor stage [68]. Oremek et al. indicated a highly significant discrimination of renal carcinoma and benign renal diseases (nephritis) based on TuM2-PK serum levels [68]. In patients with renal cell carcinomas, TuM2-PK levels were back to baseline from 6 to 11 weeks after surgical removal of the tumor when patients had no relapse, and were constantly higher in patients with relapse or metastasis [67]. Some studies report that high extracellular levels of TuM2-PK, a metabolic marker, are associated with ccRCC progression, strongly related to tumor stage and aggressivity, differentiation grade, accumulation of phosphometabolites, phospholipids and amino acids synthesis, all used by the tumoral cells [66,67,68,69,70]. Extracellular TuM2-PK stimulates tumoral increase, promotes tumoral angiogenesis, endothelial cells migration, and attachment to the extracellular matrix [69,107]. In conclusion, TK-1 and TuM2-PK factors play a key role of enzymes involved in the highly proliferative cells in advanced RCC. Various multikinase inhibitors have been developed for the treatment of renal cell carcinoma [107].

To resume, the present study is the first one in the literature that evaluated the panel of posttranslational modifications in relation to inflammation in patients with early ccRCC. However, some limitations should be noted. Our study followed patients with early ccRCC, for a period of three years. For describing PTMs as potential biomarkers in renal cancer, patients’ follow-up should be longer and more data according to cancer progression should be collected. Moreover, PTMs should be evaluated in advanced and metastatic neoplastic renal disease. Our study evaluated only PTMs in patients’ serum, but these results need to be correlated with their levels in urine for a better understanding of the physiopathological processes in tumor microenvironment.

## 4. Materials and Methods

### 4.1. Study Participants

The authors developed a prospective, observational study during a period of three years and included 55 patients with localized renal cell carcinoma and 30 heathy subjects. All the patients signed the informed consent, and all the procedures were performed according to the Declaration of Helsinki from 1975. Patients were selected from those who attended the Clinical Hospital of Nephrology “Carol Davila” and Clinical Hospital “Victor Babes”, and the study protocol was approved by the Ethics Committee of “Victor Babes Hospital” (3/18.07.2017). The groups were homogenous in terms of demographical and clinical characteristics (Table 1). We identified 107 patients with kidney tumors diagnosed by imagistic techniques (computed tomography or nuclear magnetic resonance). From those, only 71 patients had tumors of 7 cm across or smaller, with no spread to lymph nodes or distant organs (T1N0M0). The patients included in the renal cell carcinoma group were over 18-years-old, with T1N0M0. The diagnosis of clear cell renal cell carcinoma was made by histopathological exam. Based on the histological exam, 17 patients that had other types of tumors (oncocytoma, angiolipoma, papillary carcinoma, urothelial tumors) were excluded from the study. In the control group, we enrolled healthy subjects over 18-years-old, with adequate nutritional status. The exclusion criteria were: tobacco use, drug abuse, alcoholism, use of anti-inflammatory therapy (corticoids, nonsteroidal anti-inflammatories), vitamin or other supplements use, pregnancy. Patients’ characteristics are presented in Table 8.

### 4.2. Laboratory Data

The blood samples were collected from all the study participants, after 12 h of fasting, using a holder-vacutainer system. Centrifugation of the blood samples was made at 3000 g, for ten minutes, after one hour of keeping the blood at room temperature. The sera were separated and frozen at −80 degrees before analyzing. We excluded the hemolyzed, icteric, lactescent, or microbiologically contaminated samples. The samples for laboratory determinations were collected from patients in the healthy group after signing the informed consent, and in the ccRCC group, after the histopathological diagnosis of the tumor and before its surgical removal.

Circulant levels of orosomucoids were measured by immunonephelometry at 340 nm with Human reactives (MBS901995 kit) in the HumanStar300 analyzer (HUMAN Gesellschaft für Biochemica und Diagnostica mbH, Weisbaden, Germany). Sialic acid was dosed using resorcinol-chlorohydric acid. Blue chromophore was extracted with *n*-butyl/*n*-butanol acetate and spectrophotometry measured at 580 nm with the Sigma reactive (SIALICQ kit) and the BS3000 analyzer (SINNOWA Medical Science & Technology, Nanjing, China).

Nitrotyrosine (3-NT) is considered a specific biomarker for oxidative alterations mediated by peroxynitrite–antioxidant that induces the nitration of the rest of the tyrosine from proteins. Circulant 3-NT was assessed immunoenzymatically by ELISA using the R&D systems reactive (MAB3248 kit) and a TECAN analyzer (Tecan, Switzerland).

Carbonyl groups, as result of oxidative degradation of proteins, were determined by spectrophotometric methods, in reaction with 2,4-dinythrophenylhydrasine that induces hydrazone formation, using the HumanStar300 analyzer (HUMAN Gesellschaft für Biochemica und Diagnostica mbH, Weisbaden, Germany) and Merck reactives (MAK094 kit).

TDHPs were measured using a recently developed spectrophotometric method [108]. The dynamic and reducible disulfide bonds were transformed into free functional thiol groups by using sodium borohydride (NaBH4, 10 mM) according to the reaction:

**R-S2-R ‘+ NaBH4 → 2 R-SH + BH3 + Na.**

In the next step, the amount of NaBH4, which was not used in the reaction, was removed with formaldehyde (10 mM, pH 8.2). The levels of native thiol (TN) and total thiol (TT) were determined using 5,5’-dithiobis-2-nitrobenzoic acid (DTNB, 10 mM) according to the reaction:

**R-SH + DTNB → R-TNB + TNB.**

The final product, 2-nitro-5-thiobenzoate (TNB), ionized at alkaline pH and turned yellow. An automatic biochemistry analyzer (HumaSTAR 300, (HUMAN Gesellschaft für Biochemica und Diagnostica mbH, Weisbaden, Germany) and Merck reactives were used. The method allows the evaluation of functional disulfide bonds in the sample. Half of the difference between TT and NT was considered the disulfide (DS) level. The disulfide/native thiol ratio (DS/NT), disulfide/total thiol ratio (DS/TT), and native thiol/total thiol ratio (NT/TT) were calculated. TDHPs were represented by:

- NT (-SH), determined spectrophotometrically, expressed as μmol/L serum;
- TT (-SH + -S-S-), determined spectrophotometrically, expressed as μmol/L serum;
- DS (-S-S), determined by calculation, expressed as μmol/L serum;
- DS/NT (-S-S- × 100/-SH) was calculated, expressed as %;
- DS/TT (-S-S- × 100/-SH + -S-S-) was calculated, expressed as %;
- NT/TT (-SH × 100/-SH + -S-S-) was calculated, expressed as %.

Symmetric dimethylarginine was assessed by the ELISA method—competitive variant (Elabscience, USA—E-EL-0042 kit) using a TECAN analyzer (Tecan, Switzerland). The method is sensible (0.09 nmols/mL), reproducible (95–97%), repeatable (variation coefficient under 10%), specific for SDMA, with no cross reactions or interferences with others structural analogs. It has large limits of detection (0.16–10 nmols/mL), is cheap (low quantities of reactives are needed—50 µL), rapid, and adapted for varied biological samples (serum, plasma, urine, tissue, cellular lysate). The technique uses a specific primary antibody for SDMA a secondary antibody, specific for the primary antibody that is enzymatically marked. The intensity of the yellow color measured at 450 nm is inverse proportional with SDMA concentration. A high concentration of SDMA decreases the photometric signal. Sample SDMA is calculated based on the standard curve elaborated in identical experimental conditions. SDMA values are expressed in nmols/L serum.

Asymmetric dimethylarginine was assessed by the ELISA method—the competitive variant (Elabscience, USA—E-EL-0042 kit), using a TECAN analyzer (Tecan, Switzerland). The method has high sensibility and reproducibility, a large domain of concentration linearity, and the possibility to adapt to varied samples. The intensity of the yellow color measured at 450 nm is inverse proportional with ADMA concentration. Sample ADMA is calculated based on the standard curve elaborated in identical experimental conditions. ADMA values are expressed in nmols/L serum.

sIL-6R and sRAGE were assessed by the sandwich ELISA method, using immunoenzymatic kits (R&D SYSTEMS (DR600 and SRG00 kits), USA), the results being evaluated at 450 nm, using a TECAN analyzer (Tecan, Switzerland). sIL6R was dosed by immunoenzymatic method with sensitivity at 15.1 pg/mL and assay range at 31.2–2000 pg/mL. sRAGE was determined by immunoenzymatic method with sensitivity at 16.14 pg/mL and assay range at 78–5000 pg/mL.

Serum TK-1 (E.C.2.7.1.21) assessment was made by the sandwich ELISA method AroCellTK210 (Sweden), with detection range from 0.1 to 2 (μg/L) and sensitivity 0.05 (μg/L). The optic density was measured by spectrophotometry at 450 nm, using a TECAN analyzer (Tecan, Switzerland).

TuM2-PK (E.C.2.7.1.40) quantitative assessment was made by the ELISA method using a test developed by Sche. Bo. Biotech AG, Giessen, Germany with a TECAN analyzer (Tecan, Switzerland). The test is based on using two specific monoclonal antibodies against M2-PK and that do not react with L-PK, R-PK, M1-PK, and PK.

### 4.3. Statistical Analysis

The data were presented using the mean and standard deviation. The comparison of data between groups was based on *t*-tests. The relation between the markers of posttranslational modifications and inflammatory markers was assessed by Spearman’s correlation coefficient, not before the assessment of data normality by the Kolmogorov–Smirnov test. The level of significance (*p*) chosen was 0.05 (5%) and the confidence interval was 95% for hypothesis testing.

## 5. Conclusions

Many malignant disorders are characterized by PTM disturbances, molecular mechanisms that ensure a rapid and dynamic response of a biological system to intra- and extracellular stimuli. A large number of studies regarding PTM analysis in neoplasia have being developed during recent years. Our present study is the first one in the literature that evaluated a large specter of PTM and their relation with inflammation in patients with early ccRCC. Our results showed some characteristics of early ccRCC: high production of cytokines, substrate hypersialylation, induced nitrosative and carbonylic stress, arginine hypermethylation, TDH alteration, regulatory role of soluble receptors (sRAGE, sIL-6R) in RAGE and IL-6 signaling, the modulatory effect of TK-1and TuM2-PK in controlling the level of phosphometabolites in neoplastic cells. These data could be the initial point for development of a panel of biomarkers such as total sialic acid, orosomucoids, nitrotyrosine, carbonylic metabolites, ADMA, SDMA, and thiol-disulfide equilibrium for early diagnosis of ccRCC. Moreover, they could be considered a specific disease PTM signature which underlines the transition from early to advanced stages in this neoplasia, and of a therapeutic target in kidney oncogenesis.

In conclusion, PTMs are considered an efficient strategy used by the cancerous cells for their metabolism reprogramming, for unlimited proliferation, avoiding cellular death, autonomy against growth factors, suppression of growth inhibitory signals, and providing angiogenic signals, tissue invasion, tumor induced tolerance, and drug resistance.

## Figures and Tables

**Table 1:** Glycosylation status in the studied groups.

PTM	RCC (*n* = 71 Subjects)	Control (*n* = 55 Subjects)	*p* Value
Sialic acid (mg/dL)	112.78 ± 21.87	50.13 ± 3.66	<0.001
Orosomucoids (g/L)	5.72 ± 0.47	0.86 ± 0.14	<0.001

PTM—posttranslational modifications; RCC—renal cell carcinoma; *p*—statistical significance. A *t*-test was used.

**Table 2:** Protein nitration and carbonylation status in the studied groups.

PTM	RCC (*n* = 71 Subjects)	Control (*n* = 55 Subjects)	*p* Value
3-Nitrotyrosine (μmol/L)	0.335 ± 0.072	0.135 ± 0.012	<0.001
PCO (μmol/L)	45.14 ± 7.80	22.70 ± 2.23	<0.001

PTM—posttranslational modifications; RCC—renal cell carcinoma; PCO—carbonylic groups; *p*—statistical significance. A *t*-test was used.

**Table 3:** Thiol-disulfide homeostasis in the studied groups.

PTM	RCC (*n* = 71 Subjects)	Control (*n* = 55 Subjects)	*p* Value
NT (μmol/L)	360.16 ± 11.89	401.83 ± 4.71	<0.001
TT (μmol/L)	409.40 ± 12.19	440.87 ± 4.80	<0.001
DS (μmol/L)	24.62 ± 2.52	19.52 ± 0.38	<0.001
DS/NT	6.84 ± 0.76	4.85 ± 0.11	<0.001
DS/TT	6.01 ± 0.59	4.42 ± 0.09	<0.001
NT/TT	87.97 ± 1.18	91.15 ± 0.18	<0.001
GGT (U/L)	29.47 ± 6.78	27.33 ± 9.63	>0.05

PTM—posttranslational modifications; RCC—renal cell carcinoma; NT—native thiol; TT—total thiol; DS—disulfide; GGT—gamma-glutamyl-transpeptidase; *p*—statistical significance. A *t*-test was used.

**Table 4:** Protein methylation status in the studied groups.

PTM	RCC (*n* = 71 Subjects)	Control (*n* = 55 Subjects)	*p* Value
SDMA (μmol/L)	1.39 ± 0.38	0.51 ± 0.04	<0.001
ADMA (μmol/L)	0.86 ± 0.35	0.59 ± 0.06	<0.001
ADMA/SDMA ratio	0.67 ± 0.34	1.16 ± 0.11	<0.001

PTM—posttranslational modifications; RCC—renal cell carcinoma; SDMA—symmetric dimethylarginine; ADMA—asymmetric dimethylarginine; *p*—statistical significance. A *t*-test was used.

**Table 5:** Serum levels of sIL-6R and sRAGE in the studied groups.

PTM	RCC (*n* = 71 Subjects)	Control (*n* = 55 Subjects)	*p* Value
sIL-6R (ng/mL)	100.58 ± 10.21	102.47 ± 10.18	>0.05
sRAGE (pg/mL)	1001.47 ± 145.27	1014.40 ± 89.59	>0.05

PTM—posttranslational modifications; RCC—renal cell carcinoma; sIL6R—soluble receptor for Interleukin 6; sRAGE—soluble receptor for advanced glycation end products; *p*—statistical significance. A *t*-test was used.

**Table 6:** TK-1 and TuM2-PK status in the studied groups.

PTM	RCC (*n* = 71 Subjects)	Control (*n* = 55 Subjects)	*p* Value
TK-1 (ug/L)	0.19 ± 0.04	0.18 ± 0.03	>0.05
TuM2-PK (U/mL)	12.3 ± 3.4	11.6 ± 2.3	>0.05

PTM—posttranslational modifications; RCC—renal cell carcinoma; TK1—thymidine kinase 1; TuM2-PK—tumoral pyruvate-kinase 2; *p*—statistical significance. A *t*-test was used.

**Table 7:** Statistical relation between PTMs and inflammatory markers in renal cell carcinoma.

PTM		CRP	IL-6	IL-8
**Sialic acid**	*r*	**0.26**	**0.39**	**0.24**
	*p*	**0.02**	**0.01**	**0.03**
Orosomucoids	*r*	**0.24**	**0.30**	**0.25**
	*p*	**0.04**	**0.01**	**0.04**
Nitrotyrosine	*r*	0.09	**0.24**	0.11
	*p*	0.50	**0.04**	0.42
Carbonylic groups	*r*	0.08	**0.40**	0.02
	*p*	0.51	**0.003**	0.87
NT	*r*	**−0.84**	−0.07	0.09
	*p*	**<0.001**	0.28	0.18
TT	*r*	**−0.75**	**−0.18**	0.15
	*p*	**<0.001**	**0.03**	0.07
DS	*r*	0.17	−0.25	0.13
	*p*	0.20	0.06	0.30
DS/NT	*r*	**0.40**	−0.21	0.09
	*p*	**0.02**	0.09	0.46
DS/TT	*r*	**0.40**	−0.21	0.09
	*p*	**0.002**	0.11	0.46
NT/TT	*r*	**−0.40**	0.21	0.09
	*p*	**0.02**	0.10	0.46
GGT	*r*	0.15	0.03	0.05
	*p*	0.26	0.77	0.36
SDMA	*r*	0.07	0.17	0.05
	*p*	0.61	0.21	0.14
ADMA	*r*	0.05	0.05	0.70
	*p*	0.71	0.69	0.51
ADMA/SDMA	*r*	0.02	0.16	0.03
	*p*	0.86	0.25	0.78
sIL -6R	*r*	0.11	0.07	0.12
	*p*	0.40	0.59	0.37
sRAGE	*r*	0.03	0.01	0.02
	*p*	0.82	0.96	0.89
TK-1	*r*	**0.38**	0.09	0.01
	*p*	**0.04**	0.45	0.9
TuM2-PK	*r*	0.19	0.12	0.30
	*p*	0.37	0.26	0.06

PTMs—posttranslational changes; RCC—renal cell carcinoma; NT—native thiol; TT—total thiol; DS—disulfide; GGT—gamma-glutamyl-transpeptidase; ADMA—asymmetric dimethylarginine; SDMA—symmetric dimethylarginine; sIL-6R—soluble receptor for Interleukin 6; sRAGE—soluble receptor for advanced glycation end products; TK1—thymidine kinase 1; TuM2-PK—tumoral pyruvate-kinase 2; IL-6—interleukin 6; IL-8—interleukin 8; *p*—statistical significance; *r*—correlation coefficient. Spearman’s correlation coefficient was used, not before the assessment of data normality by Kolmogorov–Smirnov test.

**Table 8:** Patients’ characteristics in the studied groups.

Characteristics	RCC (*n* = 71 Subjects)	Control (*n* = 55 Subjects)	*p* Value
Women:Men ratio	1/1.25	1/1	NS
Age (years old)	58.6 ± 11.3	57.8 ± 9.5	NS
BMI (Kg/mp)	22.9 ± 1.7	23.0 ± 2.1	NS
Systolic Pressure (mmHg)	11.9 ± 1.3	11.2 ± 1.1	NS
Diastolic pressure (mmHg)	6.1 ± 0.7	6.0 ± 0.5	NS
Leucocytes (cells/mmc)	6500 ± 2008	6050 ± 1040	NS
Erythrocytes (10^3^ cells/mmc)	4950 ± 317	5010 ±602	NS
Phosphorus (mg/dL)	3.7 ± 0.8	3.6 ± 0.9	NS
Calcium (mg/dL)	9.22 ± 0.4	9.14 ± 0.5	NS
LDH(U/L)	299 ± 65	301 ± 61	NS
Glycemia (mg/dL)	81.1 ± 10.3	79.9 ± 12.7	NS
Urea (mg/dL)_	33.6 ± 11.4	34.1 ± 0.9	NS
Creatinine (mg/dL)	1.01 ± 0.12	0.78 ± 0.11	NS
eGFR (ml/min/1,73 mp)	89.45 ± 2.43	95.17 ± 3.12	0,05
Uric acid (mg/dL)	4.1 ± 1.3	3.9 ± 1.0	NS
ASAT (U/L)	19.4 ± 10.2	17.0 ± 8.5	NS
ALAT (U/L)	18.1 ± 13.2	16.2 ± 8.2	NS
Cholesterol (mg/dL)	142.5 ± 22.3	147.2 ± 23.5	NS
Triglycerides (mg/dL)	89.5 ± 12.7	81.4 ± 10.5	NS
Albumin (g/dl)	3.66 ± 0.25	3.98 ± 0.34	<0.05
UACR (mg/g creatinine)	12.81 ± 3.88	8.84 ± 2.97	0.057
ESR (mm/h)	12.7 ± 10.1	5.5 ± 5.0	0.048
CRP (mg/dL)	1.84 ± 0.62	0.29 ± 0.15	<0.001
IL-6 (pg/mL)	9,13 ± 2.95	3.41 ± 0,59	<0.001
IL-8 (pg/mL)	59.31 ± 18.75	10.14 ± 2.83	<0.001

RCC—renal cell carcinoma; BMI—body mass index; LDH—lactatdehydrogenase; eGFR— estimated glomerular filtration rate; ASAT—aspartate aminotransferase; ALAT—alanyl aminotransferase; UACR—urinary albumin: creatinine ratio; ESR—erythrocyte sedimentation rate; CRP—C reactive protein; IL-6—interleukin 6; IL-8—interleukin 8; *p*—statistical significance. A *t*-test was used.

**Figure A1:**
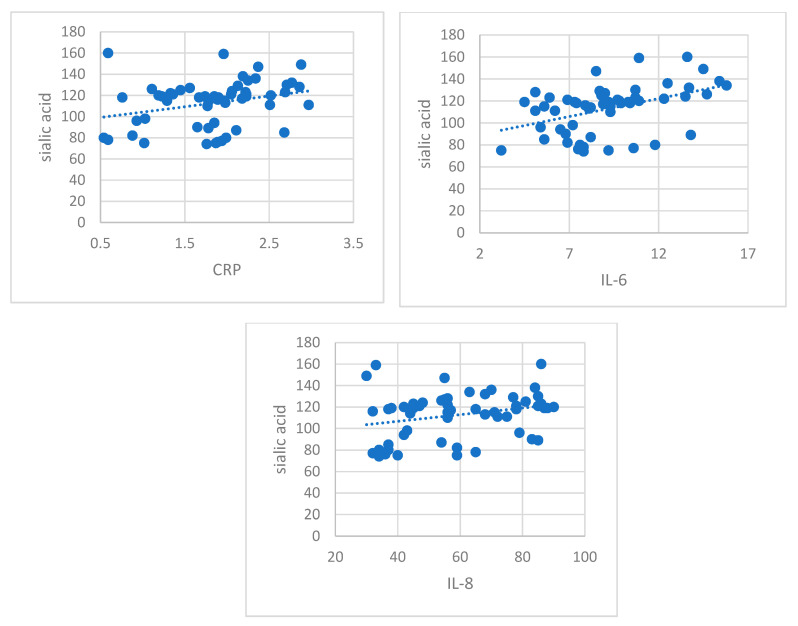
Sialic acid and CRP, IL-6, and IL-8.

**Figure A2:**
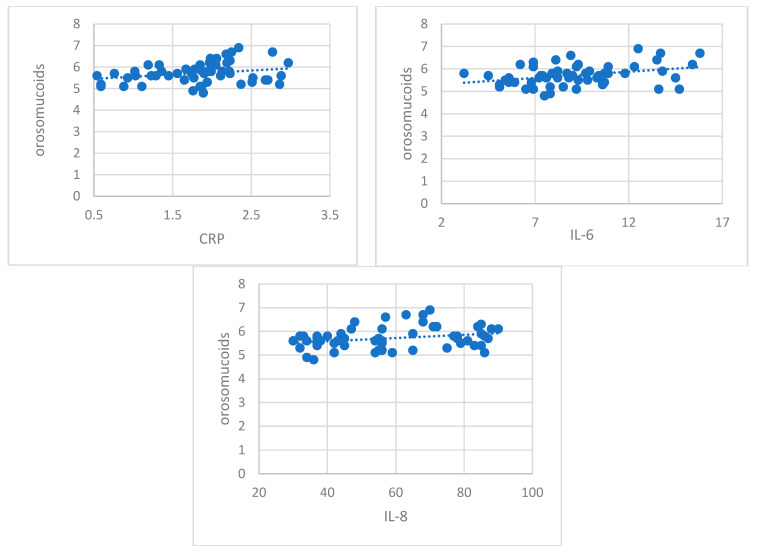
Orosomucoids and CRP, IL-6, and IL-8.

**Figure A3:**
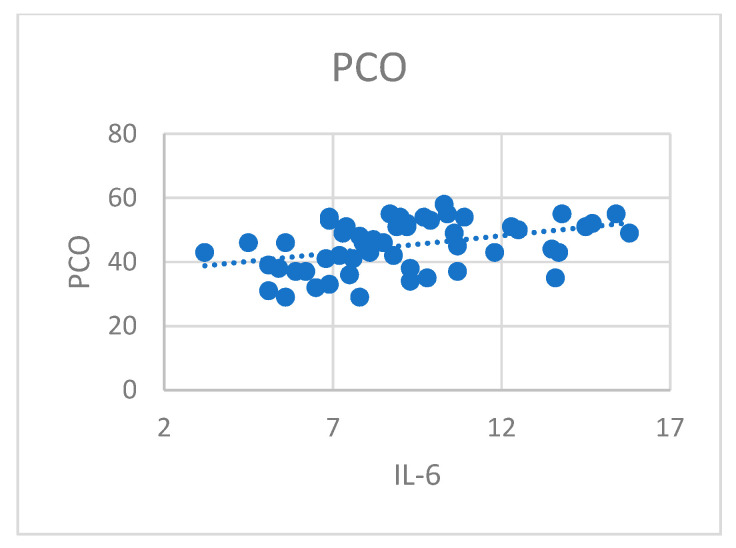
PCO and IL-6.

**Figure A4:**
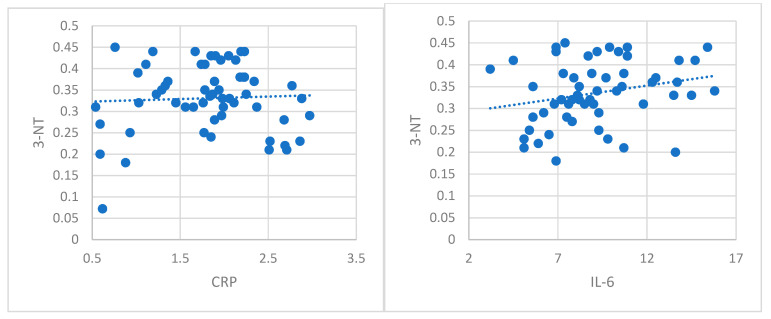
3-NT and IL-6 and CRP.

**Figure A5:**
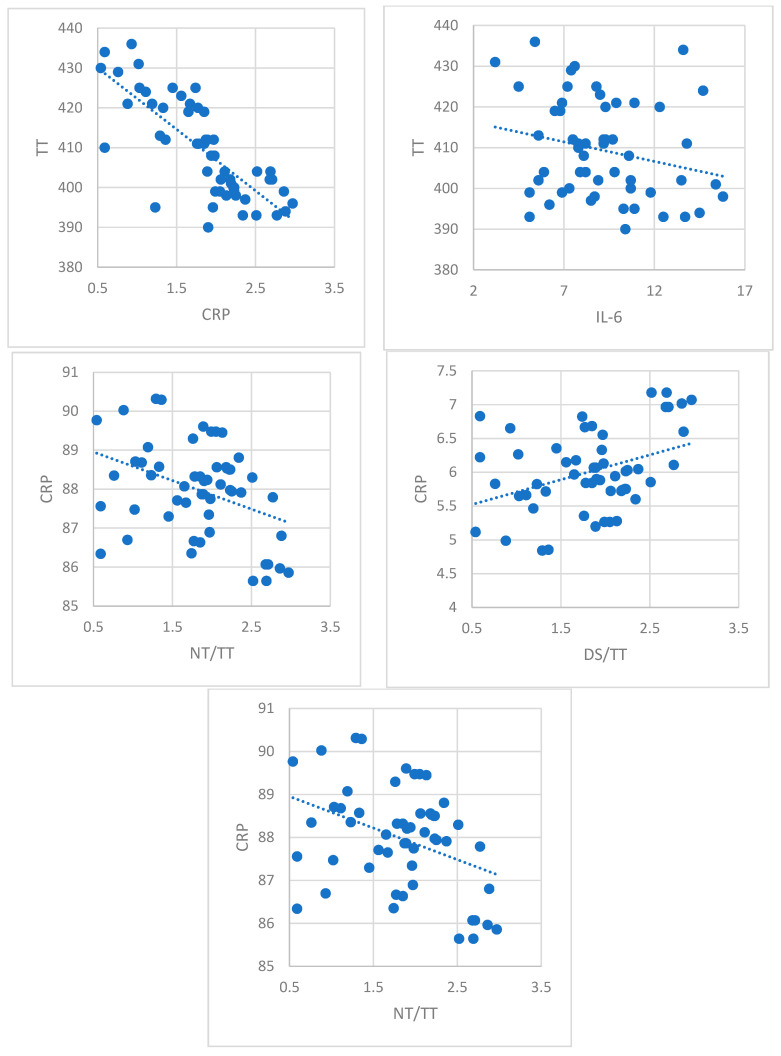
Thiol/Disulfides and CRP and IL-6.

## Appendix A

Statistical Relation between PTM and Inflammatory Markers in Renal Cell Carcinoma.
